# Supplementary material for: [89Zr]-Pertuzumab PET Imaging Reveals Paclitaxel Treatment Efficacy Is Positively Correlated with HER2 Expression in Human Breast Cancer Xenograft Mouse Models
Source: Molecules. 2021 Mar 12;26(6):1568. doi: 10.3390/molecules26061568 (PMC8001650; doi:10.3390/molecules26061568)
Supplement: Supplementary file 1 [file molecules-26-01568-s001.pdf]

Supplemental Figures

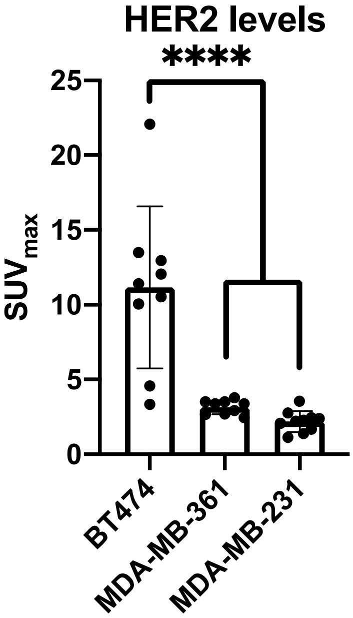

Figure S1. Standard uptake value (SUV) maximum (SUV_{max}) of of [^{89}Zr]-pertuzumab PET in two HER2+ (BT474, MDA-MB-361) and one HER2- (MDA-MB-231) tumor model. ****, p<0.0001.

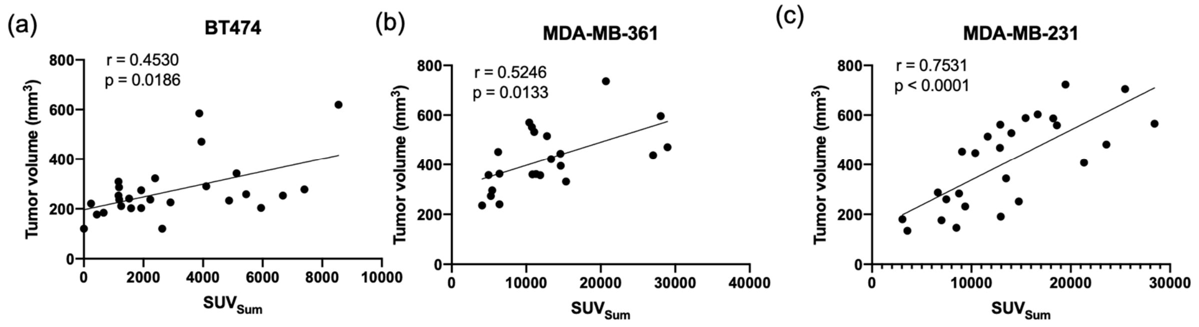

Figure S2. [^{18}F]-FDG PET imaging reveals early cellular signaling of therapeutic response prior to changes in tumor volume. Sum of standard uptake value (SUV) of [^{18}F]-FDG PET is correlated with tumor volume measured by caliper in BT474 (**a**), MDA-MB-361 (**b**), and MDA-MB-231 (**c**). Spearman's correlation: MDA-MB-231 (r=0.7531, p<0.0001), MDA-MB-361 (r=0.5246, p=0.0133), BT474 (r=0.4530, p=0.0186).

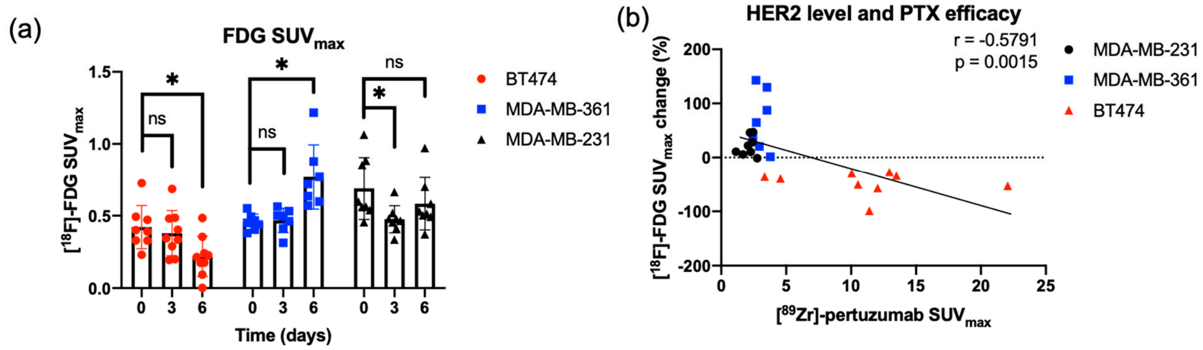

Figure S3. HER2 expression level is positively correlated with paclitaxel treatment efficacy. (**a**) SUV$_{max}$ of [^{18}F]-FDG in BT474, MDA-MB-361, and MDA-MB-231 tumors from day 0 to day 6. (ANOVA and Tukey's multiple comparisons test: ns, non-significant, p<0.05). (**b**) The percentage changes of SUV$_{max}$ of [^{18}F]-FDG from day 3 to day 6 are negatively correlated with SUV$_{max}$ of [^{89}Zr]-pertuzumab (Spearman's correlation: r=-0.5791, p=0.0015).

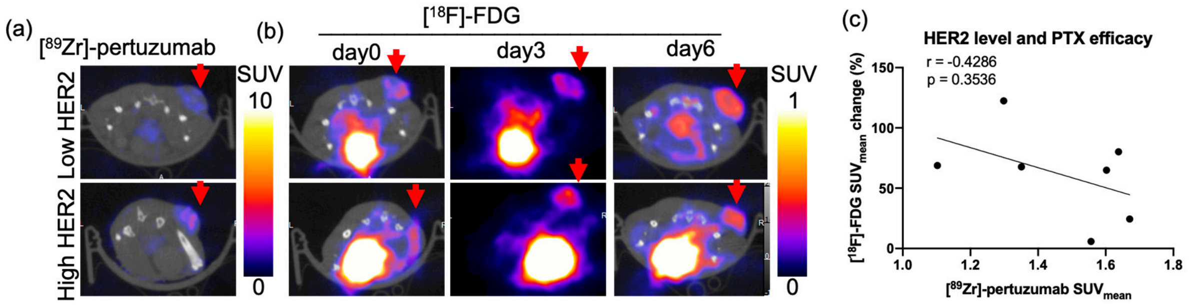

Figure S4. HER2 expression level is correlated with paclitaxel treatment efficacy in MDA-MB-361 tumor model. (**a**) Representative [^{89}Zr]-pertuzumab PET images of MDA-MB-361 tumors with high and low HER2 levels in transverse section. Red arrows point at tumors. (**b**) Representative [^{18}F]-FDG PET images of MDA-MB-361 tumors with high and low HER2 levels from day 0 to day 6 in transverse section. Red arrows point at tumors. (**c**) The percentage changes of SUV$_{mean}$ of [^{18}F]-FDG from day 0 to day 6 are negatively correlated with SUV$_{mean}$ of [^{89}Zr]-pertuzumab in MDA-MB-361 tumors (Kendall tau correlation: r=-0.3333, p=0.2595).
